# SARS-CoV-2 RT-qPCR Ct values in saliva and nasopharyngeal swab samples for disease severity prediction

**DOI:** 10.1080/20002297.2023.2213106

**Published:** 2023-05-17

**Authors:** Kristina Snipaitiene, Birute Zablockiene, Rasa Sabaliauskaite, Kristina Zukauskaite, Elzbieta Matulyte, Tautvile Smalinskaite, Mindaugas Paulauskas, Rolandas Zablockis, Mantvydas Lopeta, Julius Gagilas, Alina Puriene, Ligita Jancoriene, Sonata Jarmalaite

**Affiliations:** a Institute of Biomedical Sciences, Life Sciences Center, Vilnius University, Vilnius, Lithuania; b Laboratory of Genetic Diagnostic, National Cancer Institute of Lithuania, Vilnius, Lithuania; c Center of Infectious Diseases, Vilnius University Hospital Santaros Klinikos, Vilnius, Lithuania; d Clinic of Infectious Diseases and Dermatovenerology, Institute of Clinical Medicine, Faculty of Medicine, Vilnius University, Vilnius, Lithuania; e Faculty of Medicine, Vilnius University, Vilnius, Lithuania; f Clinic of Chest Diseases, Immunology and Allergology, Institute of Clinical Medicine, Faculty of Medicine, Vilnius University, Vilnius, Lithuania; g Centre of Pulmonology and Allergology, Vilnius University Hospital Santaros Klinikos, Vilnius, Lithuania; h JSC Diagnolita, Vilnius, Lithuania; i Institute of Odontology, Faculty of Medicine, Vilnius University, Vilnius, Lithuania

**Keywords:** SARS-CoV-2, COVID-19, severity, saliva, RT-qPCR, Ct values, viral dynamics

## Abstract

**Background:**

Comparison of clinical value of RT-qPCR-based SARS-CoV-2 tests performed on saliva samples (SSs) and nasopharyngeal swab samples (NPSs) for prediction of the COVID-19 disease severity.

**Methods:**

Three paired SSs and NPSs collected every 3 days from 100 hospitalised COVID-19 patients during 2020 Jul-2021 Jan were tested by RT-qPCR for the original SARS-CoV-2 virus and compared to 150 healthy controls. Cases were divided into mild+moderate (Cohort I, *N* = 47) and severe disease (Cohort II, *N* = 53) cohorts and compared.

**Results:**

SARS-CoV-2 was detected in 65% (91/140) vs. 53% (82/156) of NPSs and 49% (68/139) vs. 48% (75/157) of SSs collected from Cohort I and II, respectively, resulting in the total respective detection rates of 58% (173/296) vs. 48% (143/296) (*P* = 0.017). Ct values of SSs were lower than those of NPSs (mean Ct = 28.01 vs. 30.07, *P* = 0.002). Although Ct values of the first SSs were significantly lower in Cohort I than in Cohort II (*P* = 0.04), it became negative earlier (mean 11.7 vs. 14.8 days, *P* = 0.005). Multivariate Cox proportional hazards regression analysis showed that Ct value ≤30 from SSs was the independent predictor for severe COVID-19 (HR = 10.06, 95% CI: 1.84–55.14, *P* = 0.008).

**Conclusion:**

Salivary RT-qPCR testing is suitable for SARS-CoV-2 infection control, while simple measurement of Ct values can assist in prediction of COVID-19 severity.

## Introduction

Severe acute respiratory syndrome coronavirus 2 (SARS-CoV-2) is a novel coronavirus that emerged in late 2019 and spread across the globe, with the number of newly confirmed cases growing to more than 760 million [1]. Nasopharyngeal specimen (NPS) quantitative reverse transcription PCR (RT-qPCR) is still considered the gold-standard laboratory technique for the etiological diagnosis of SARS-CoV-2 infection, having the highest sensitivity of all upper respiratory tract specimens [2,3]. However, collecting NPSs is technically challenging, and there is a risk of droplet transmission of the infection to healthcare professionals [4]. In addition, the number of false-negative results depends on the quality of the sample, sensitivity and specificity of the used reagents and on the experience and training of the person performing the procedure as well [5]. Multiple studies support non-invasive specimen collection from saliva as a suitable alternative to NPSs [6–11]. Saliva specimens are already used in some countries for pandemic control as a new gold-standard sample type [12]. However, the clinical application of the cycle threshold (Ct) values of saliva and NPS samples is understudied, and definitive data to support the prediction of disease severity is lacking [13]. Quantitative changes of viral SARS-CoV-2 RNA at different stages of disease could provide clinically significant information regarding the severity of the disease [14]. Hence, viral load changes might serve as a guideline for therapy prescription and impact clinical outcomes of the disease [15].

In this study, we compared RT-qPCR measures of paired and serial SARS-CoV-2 nasopharyngeal and saliva swab samples to assess the prognostic test value and on predicting the COVID-19 disease severity.

## Materials and methods

### Participant cohorts

NPSs and corresponding SSs were collected from 100 COVID-19 patients treated at the Center of Infectious Diseases of Vilnius University Hospital Santaros Klinikos (VUHSK, Vilnius, Lithuania) from 2020 July to 2021 January for the original SARS-CoV-2 testing. For the control group, NPSs and corresponding SSs were collected from 150 persons visiting VUHSK for routine procedures (drug prescriptions, vaccines, etc.). Criteria for inclusion of the COVID-19 patient’s group were as follows: persons over the age of 18; a diagnosis of COVID-19 has been established and are being treated in an inpatient facility. Criteria for inclusion of the control group in the study were as follows: persons over the age of 18; not diagnosed with COVID-19. Exclusion criteria for COVID-19 patients and control groups were as follows: pregnant women; persons under the age of 18; persons with mental disabilities; persons suffering from oncological diseases.

This was a prospective, observational study. The primary outcome was the ability of salivary SARS-CoV-2 RNA RT-qPCR testing to predict the severity of COVID-19. The primary predictive variable was salivary SARS-CoV-2 RNA RT-qPCR test Ct values, dichotomized into Ct ≤ 30 and Ct > 30. The secondary outcomes were suitability of salivary SARS-CoV-2 RNA RT-qPCR test to detect severe COVID-19, and time till SARS-CoV-2 virus clearance, defined as duration of positive salivary or nasopharyngeal SARS-CoV-2 RNA RT-qPCR test.

The bioethics approval was obtained by the Regional Bioethics Committee (No. 2020/7-N4˗1245˗725), and the study was conducted following the Declaration of Helsinki. The informed consent forms were gained from all the participants before the beginning of the study. The SARS-CoV-2 infection was confirmed by RT-qPCR from NPS before the hospitalization at VUHSK. According to the disease severity, patients were dichotomized into two cohorts: mild and moderate disease cases (*N* = 47, Cohort I) and severe disease cases (*N* = 53, Cohort II). The patients were divided into groups dependent on the disease severity according to the National Institutes of Health guidelines [16], which briefly define the severe cases as individuals having an oxygen saturation measured by pulse oximetry on room air at sea level (SpO_2_) <94%, a ratio of arterial partial pressure of oxygen to fraction of inspired oxygen (PaO_2_/FiO_2_) <300 mm Hg, and a respiratory rate >30 breaths/min, or lung infiltrates >50%; moderate – SpO_2_≥94% and evidence of lower respiratory disease during clinical assessment or imaging; mild – any of the various signs and symptoms of COVID-19 (e.g. fever, cough, sore throat, malaise, headache, muscle pain, nausea, vomiting, diarrhoea, loss of taste and smell) but do not have shortness of breath, dyspnoea, or abnormal chest imaging.

### Sample collection

NPSs were collected by following manufacturers’ protocols and hospital’s procedures. At least 2 mL of saliva samples from 50 patients were collected into sterile tubes, whereas 2 mL saliva samples from another 50 patients and 150 controls were taken by using a Saliva Collection Kit (#SCK, Diagnolita, Vilnius, Lithuania) and mixed with 2 mL stabilizing media immediately after the collection as described in recent publication [11]. All the samples were stored at −20°C until use.

When possible, two subsequent SS and NPS collections from the same hospitalized patients were performed 3 days apart. Altogether, 296 infectious NPSs (I 100, II 97, III 99) and 296 SSs (I 99, II 98, III 99) were taken, in total assembling a collection of 892 samples (592 infectious, 300 healthy).

### RNA extraction

Total RNA was extracted by using Saliva NA Purification Kit (#SPK, Diagnolita) and following the manufacturer’s protocol. Briefly, the purification solution, consisting of stabilization media, binding solution, magnetic beads, reducing reagent, and proteinase K, was mixed with 300 µL of nasopharyngeal swab medium or 250 µL of unstabilized saliva (490 µL of the saliva-stabilizer mix for stabilized saliva samples) and incubated in thermoshaker for 10 min at 65°C temperature and 900 rpm [11]. After two washing steps with PB1 and PB2 buffers (Diagnolita), the residual ethanol was eliminated by heating. Purified nucleic acids were eluted into 50 µL elution buffer. Eluted nucleic acids were immediately used for downstream applications or stored at −20 until use.

### cDNA synthesis and quantitative reverse transcription PCR (RT-qPCR)

Five microlitre of total RNA was used for one-step cDNA synthesis and RT-qPCR reaction in a total volume of 20 µL consisting of 20× Primer-Probe Mix for SARS-CoV-2 Detection (#PPM, Diagnolita) [11], 4× TaqMan Fast Virus 1-Step Master Mix (Thermo Fisher Scientific (TFS), Waltham, MA, USA), and RNase-free water. For cDNA synthesis, reaction mixes were incubated at 50°C for 5 min, and the subsequent RT-qPCR runs consisted of enzyme activation at 95°C for 20 s and 40 cycles of 95°C for 3 s and 60°C for 30 s. RT-qPCR reactions were run on the QuantStudio 5 Real-Time PCR System by using QuantStudio Design & Analysis Software v1.4.1 (both from Applied Biosystems, TFS).

### Statistical analysis

Continuous variables are presented as means and standard deviation (SD) or as median and interquartile range (IQR). Categorical variables are reported as numbers and percentages (%). Differences in characteristics among the illness severity groups were tested using Student‘s t-test for normally distributed data and using Mann–Whitney U-test for non-normally distributed data. Paired Student’s t-test was used to compare characteristics of two related groups. For categorical variables, the Pearson‘s chi-squared test or Fisher’s exact test was used. Pearson linear or Spearman rank (for ordinal variables and as a more robust measure for interval variables with highly influential points) correlation coefficients were used to find the associations between clinical, laboratory characteristics and SARS-CoV-2 RT-qPCR Cycle threshold (Ct) values. FDR correction was applied for the analyses that involved multiple comparisons to adjust the resulting P-values. Cox regression analysis was used to determine which factors had a significant impact for prognosis of severe COVID-19 infection. The Kaplan–Meier method (log-rank testing) was used to predict the positivity for SARS-CoV-2. A P-value ≤ 0.05 was defined as statistically significant. Data were analysed using the Statistical Package for Social Science software version 21.0 for Windows (IBM Corp., Armonk, NY, USA). Graphs were depicted by using GraphPad Prism v8.0.1 (GraphPad Software, Inc., San Diego, CA, USA).

## Results

### Demographics and clinical profiles

Demographic and clinical characteristics of patients according to the disease severity (Cohort I *vs*. Cohort II) are presented in Tables 1 and 2. The mean age was 56.7 ± 13.2 years (range: 25–90 years), 52.0% were male. Cohort II patients were significantly older (mean: 61.6 *vs*. 51.2 years, *P* < 0.001), had higher National Early Warning (NEW) score on admission (median: 3 *vs*. 1, *P* < 0.001) and bigger Charlson comorbidity index (median: 2 *vs*. 1, *P* < 0.001). Treatment with dexamethasone (20 *vs*. 8 patients, *P* = 0.020); dexamethasone and remdesivir combination (28 *vs*. 12 patients, P*=*0.043) or antibacterial treatment (47 *vs*. 22 patients, *P* < 0.001) was applied more often for Cohort II patients as well. Three Cohort II patients were treated with a high flow nasal cannula, one patient needed intubation and invasive mechanical ventilation and three patients died. More patients in Cohort I were included in study with a disease duration ≤10 days, while more patients in Cohort II were included in the study with a disease duration >10 days (*P* = 0.001). The median duration from symptoms onset to tests was significantly longer in Cohort II group compared to Cohort I (first sample median: 11 *vs*. 8 days, *P* = 0.003).

**Table 1.** The clinical characteristics, laboratory tests, treatment, and outcomes of patients with COVID-19 infection depending on the severity of the disease.

Characteristics	Cohort I (*N* = 47)	Cohort II (*N* = 53)	P-value
**Age, yr**			
Mean ± SD	51.2 ± 13.6	61.6 ± 10.7	**<0.001**
<65, n (%)	41 (87.2)	35 (66.0)	**0.033**
≥65, n (%)	6 (12.8)	18 (34.0)	
**Gender, n (%)**			
Male	27 (57.4)	25 (47.2)	0.364
Female	20 (42.6)	28 (52.8)	
**NEW score**			
Median (IQR)	1 (0–2)	3 (2–5)	**<0.001**
0–4, n (%)	45 (95.7)	39 (73.6)	**0.008**
≥5, n (%)	2 (4.3)	14 (26.4)	
**Charlson comorbidity index**			
Median (IQR)	1 (0–2)	2 (1–3)	**<0.001**
0–2, n (%)	42 (89.4)	31 (73.6)	**0.003**
≥3, n (%)	5 (10.6)	22 (41.6)	
**Interleukin-6, pg/ml**			
Median (IQR)	16.9 (9.2–30.2)	43.5 (22.1–67.6)	**0.001**
≤55, n (%)	39 (88.6)	34 (66.7)	**0.029**
>55, n (%)	5 (11.4)	17 (33.3)	
**Ferritin, ng/ml**			
Median (IQR)	270.2 (139.2–529.7)	652 (269–1096)	**0.010**
≤500, n (%)	35 (74.5)	22 (41.5)	**0.004**
>500, n (%)	12 (25.5)	31 (58.5)	
**CRP, mg/dl**			
Median (IQR)	25.7 (7.67–82.6)	102.5 (54.6–189.1)	**<0.001**
≤100, n (%)	39 (83.0)	26 (49.1)	**0.003**
>100, n (%)	8 (17.0)	27 (50.9)	
**WBC count, per mm^3^**			
Median (IQR)	4.9 (3.6–7.4)	7.2 (5.5–9.2)	**0.003**
≤6.0, n (%)	30 (63.8)	19 (35.8)	**0.005**
>6.0, n (%)	17 (36.2)	34 (64.2)	
**Neutrophil count, per mm^3^**			
Median (IQR)	3.4 (2.09–5.1)	5.2 (4.1–8.1)	**<0.001**
≤4.0, n (%)	29 (61.7)	11 (20.8)	**<0.001**
>4.0, n (%)	18 (38.3)	42 (79.2)	
**NLR**			
Median (IQR)	2.6 (1.9–4.6)	6.9 (3.3–8.9)	**<0.001**
≤5.94, n (%)	42 (89.4)	24 (45.3)	**<0.001**
>5.94, n (%)	5 (10.6)	29 (54.7)	
**Lymphocyte count, per mm^3^**			
Median (IQR)	1.2 (0.8–1.6)	0.9 (0.8–219)	0.055
≤1.1, n (%)	21 (44.7)	34 (64.2)	**0.040**
>1.1, n (%)	26 (55.3)	19 (35.8)	
**D-dimer, ng/ml**			
Median (IQR)	285 (150–495)	465 (350–815)	**0.001**
≤500, n (%)	36 (76.6)	29 (54.7)	**0.018**
>500, n (%)	11 (23.4)	24 (45.3)	
**LDH, IU/l**			
Median (IQR)	238 (192.5–302.5)	342 (280–420)	**<0.001**
≤245, n (%)	24 (51.1)	8 (15.1)	**<0.001**
>245, n (%)	23 (48.9)	45 (84.9)	
**Duration of fever, days**			
Median (IQR)	9 (6–13.75)	11.5 (8–17.25)	0.055
**Therapies administered, n (%)**			
Remdesivir	5 (10.6)	2 (3.8)	0.298
Dexamethasone	8 (25.5)	20 (52.8)	**0.020**
Remdesivir and dexamethasone	12 (17.0)	28 (37.7)	**0.043**
Antibiotics	22 (46.8)	47 (88.7)	**<0.001**
**Respiratory support, n (%)**			
Oxygen only	23 (48.9)	49 (92.4)	**<0.001**
HFNC	0.0 (0.0)	3 (5.7)	0.245
Invasive mechanical ventilation	0.0 (0.0)	1 (1.9)	0.530
**Length of hospitalization, days**			
Median (IQR)	11 (10–15.5)	12 (10–17)	0.163
**Death, n (%)**	0.0 (0.0)	3 (5.7)	0.245

Data are presented as mean (SD), median (IQR), and n (%). Cases with mild to moderate disease (Cohort I) and severe disease (Cohort II). Significant values are shown in bold.

Abbreviations: CRP, C-reactive protein; HFNC, high flow nasal Cannula; IQR, interquartile range; LDH, lactate dehydrogenase; NEW, National Early Warning; NLR, neutrophil to lymphocyte ratio; SD, standard deviation; WBC, white blood cell.

**Table 2.** The results of nasopharyngeal swab and saliva sample tests for SARS-CoV-2 infection depending on the severity of the disease.

Characteristics	Cohort I (*N* = 47)	Cohort II (*N* = 53)	P-value
**Median no. of days since onset of symptoms to first SS and NPS tests**	8 (5.5–11)	11 (9–13)	**0.003**
**Mean Ct value from SSs**			
First test	25.5 ± 4.2	28.0 ± 5.6	0.055
Second test	28.3 ± 3.9	28.9 ± 4.4	0.671
Third test	29.1 ± 3.3	29.5 ± 5.9	0.801
**Mean Ct value from NPSs**			
First test	28.4 ± 5.7	29.3 ± 5.1	0.570
Second test	29.8 ± 4.7	30.8 ± 4.7	0.509
Third test	31.1 ± 3.4	32.6 ± 4.9	0.055
**Ct values from the first SSs, n (%)**			
≤30,	25 (89.3)	19 (51.4)	**0.001**
>30	3 (10.7)	18 (48.6)	
**Ct values from the first NPSs, n (%)**			
≤30	22 (55.0)	16 (44.4)	0.491
>30	18 (45.0)	20 (55.6)	
**Mean no. of days since SARS-CoV-2 test conversion**			
SS	11.7 ± 3.9	14.8 ± 4.0	**0.005**
NPS	13.4 ± 4.4	14.6 ± 4.6	0.395
**The negative RT-qPCR tests for SARS-CoV-2 at the end of the study, n (%)**			
SS	29 (61.7)	35 (66.0)	0.692
NPS	26 (55.3)	27 (50.9)	0.692
**Days from the onset of symptoms to the first tests, n (%)**			
≤10	32 (68.1)	18 (34.0)	**0.001**
>10	15 (31.9)	35 (66.0)	

Data are presented as median (IQR), and n (%). Cases with mild to moderate disease (Cohort I) and severe disease (Cohort II). Significant values are shown in bold.

Abbreviations: Ct, Cycle threshold; IQR, Interquartile range; NPSs, Nasopharyngeal Swabs; SSs, Saliva specimens.

### Laboratory tests profiles

On admission, Cohort II patients had a higher concentration of interleukin-6 (IL-6, median: 43.5 *vs*. 16.9 pg/ml, *P* = 0.001), ferritin (median: 652 *vs*. 270.2 ng/ml, *P* = 0.010), C-reactive protein (median: 102.5 *vs*. 25.7 mg/dl, *P* < 0.001), D-dimer (median: 465 *vs*. 285 ng/ml, *P* = 0.001), lactate dehydrogenase (median: 342 *vs*. 238 IU/l, *P* < 0.001) compared to Cohort I patients. There were significant changes in leukocyte counts between these groups with a bigger leukocytosis (median count: 7.2 *vs*. 4.9 per mm^3^, *P* = 0.003), neutrophilia (median count: 5.2 *vs*. 3.4 per mm^3^, *P* < 0.001) and lymphopenia less than 1.1 per mm^3^ (64.2% *vs*. 44.7%, *P* = 0.040) in Cohort II patients. The neutrophil to lymphocyte ratios were significantly higher in these patients too (median: 6.9 *vs*. 2.6, *P* < 0.001).

### Nasopharyngeal swabs and saliva samples profiles

**Figure 1.**
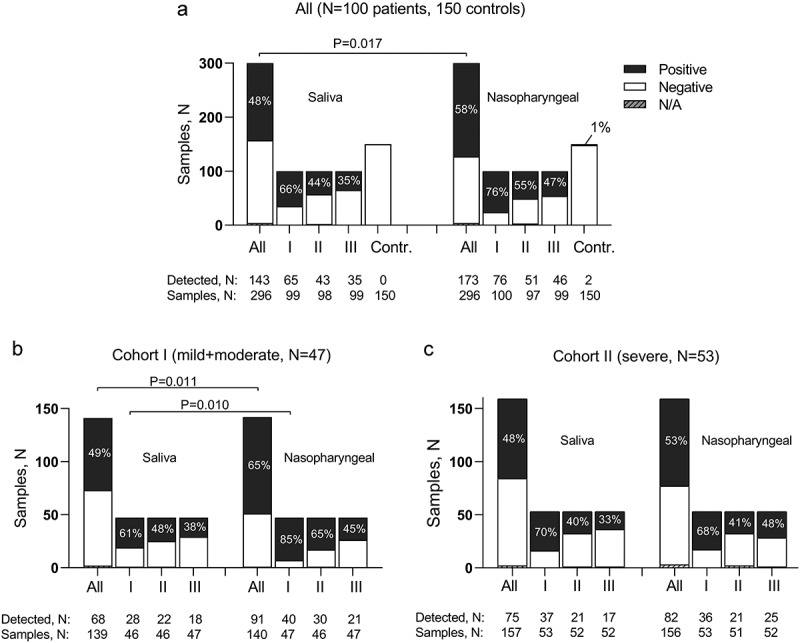
Detection rates of SARS-CoV-2 in saliva and nasopharyngeal samples collected from COVID-19 patients and controls (a) and in patients dichotomised into mild/moderate (Cohort I, b) and severe cases (Cohort II, c).

In total, SARS-CoV-2 was detected in 53% (316/592) of the samples. Out of the 296 tested NPSs and 296 SSs, significantly more SARS-CoV-2 positive samples were in NPS group − 173 (58%) *vs*. 143 (48%), respectively; *P* = 0.017 (Figure 1a). Analysing according to the cohorts, the same difference was found in Cohort I (*P* = 0.011) and was especially evident on the first RT-qPCR test (*P* = 0.010) (Figure 1b). For the comparison, NPSs and SSs collected from 150 healthy persons were tested and SARS-CoV-2 was detected in 2 NPS and none of SS samples, reaching the specificity of 99% and 100%, respectively.

**Figure 2.**
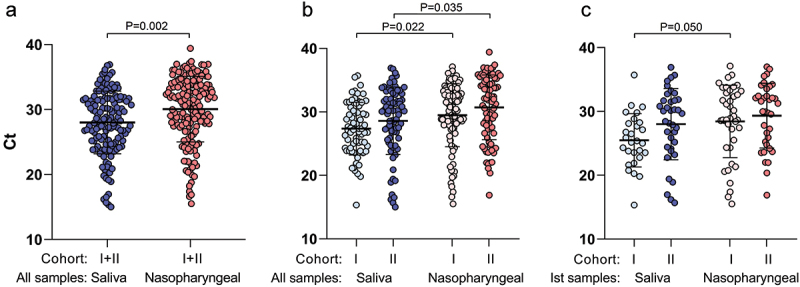
Comparison of Cycle threshold (Ct) values from positive saliva specimens and nasopharyngeal swabs between all samples altogether (a), all samples dichotomised into Cohort I vs. Cohort II, (b) and the first test only (c).

RT-qPCR test Ct values of SSs were significantly lower (reflecting higher viral load) than in NPS samples (mean 28.01 *vs*. 30.07, *P* = 0.002, Figure 2a), and the significant differences between SSs and NPSs were retained when the samples were dichotomised into Cohort I and Cohort II (*P* =0.022 and *P* = 0.035, respectively, Figure 2b), or the Ist test was analysed only (*P* = 0.05, Figure 2c). No significant differences in the mean Ct values were found between the cohorts using SS or NPT samples (*P* > 0.05, Figure 2b,c).

**Figure 3.**
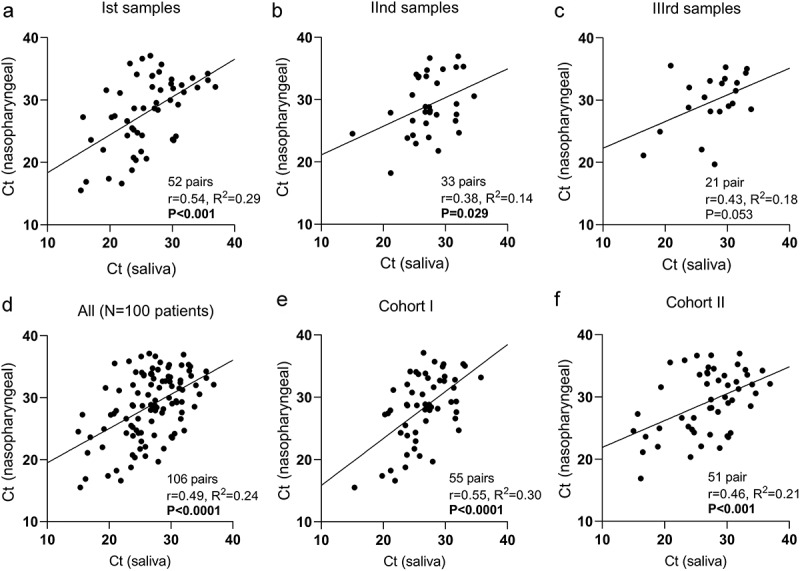
Correlation analysis of cycle threshold (Ct) values from positive saliva specimens and nasopharyngeal swabs: corresponding first (a), second (b), and third (c) sample tests, altogether (d) and according to cohorts (e and f).

The correlation analysis of paired NPS and SS test results for SARS-CoV-2 repeated three times every 3 days was performed. A statistically significant moderately strong correlation on the first (*r* = 0.54, *P* < 0.001, Figure 3a) and weak correlation on the second (*r* = 0.38, *P* = 0.029, Figure 3b) tests between the Ct values of SSs and NPSs was observed; however, there was no significant correlation on the third test (Figure 3c). Significant correlations were found between all SS and NPT samples and between Cohort I and Cohort II samples analysed separately as well (Figure 3d–f).

### Virus dynamics over time

**Figure 4.**
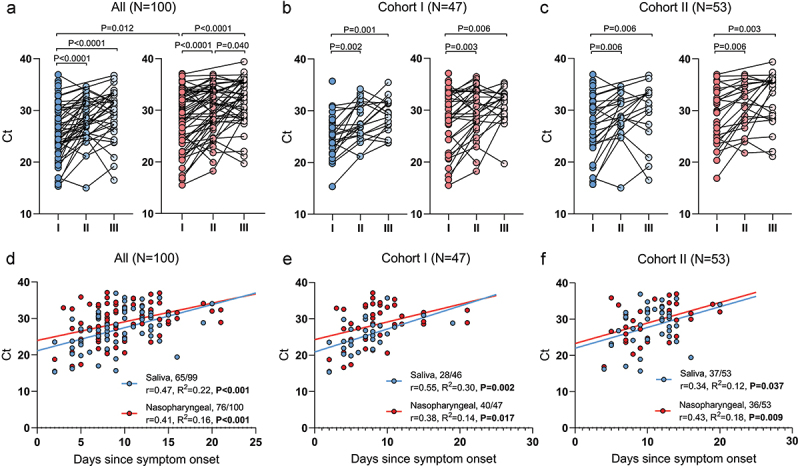
Analysis of serial saliva and nasopharyngeal samples: comparison of cycle threshold (Ct) values (a–c) and correlation of Ct values from saliva, nasopharyngeal and days since onset of symptoms (d–f). r, Pearson correlation.

Analysis of virus detection dynamics over time confirmed that virus loads reflected by lower Ct values in both, NPSs and SSs were higher in the Ist samples and gradually decreased during time in the second and third tests (*P* < 0.05; Figure 4a). Separate cohort analysis showed the same tendencies (Figure 4b,c). Also, the correlation analysis between Ct values from the first SSs and NPSs and days since the onset of symptoms was done (Figure 4d–f). There was a significant and moderately strong correlation between SSs SARS-CoV-2 RT-qPCR Ct and duration of the disease (*r* = 0.47, *P* < 0.001), as well as in the case of NPSs (*r* = 0.41, *P* < 0.001). In Cohort I, a stronger correlation was noticed for SS samples than for NPTs (*r* = 0.55, *P* = 0.002 vs. *r* = 0.38, *P* = 0.017); however, in Cohort II, vice versa association was found (*r* = 0.34, *P* = 0.037 vs. *r* = 0.43, *P* = 0.009). SARS-CoV-2 RT-qPCR test became negative earlier in SSs in Cohort I as compared to Cohort II (mean 11.7 vs. 14.8 days, *P* = 0.005), while there was no such difference in NPS tests.

### SARS-CoV-2 Ct values association with disease severity markers

SSs (*N* = 61) and NPSs (*N* = 73) first tests’ SARS-CoV-2 RT-qPCR Ct values correlations with clinical and laboratory characteristics are presented in Table 3. There was a strong positive correlation between the number of days since the onset of symptoms and Ct values in both saliva and nasopharyngeal analyses (*P* < 0.001). Also, Ct values of SSs correlated with NEW score, ferritin, CRP, D-dimer concentrations, WBC, neutrophil and lymphocyte counts, whereas NPSs Ct values correlated with CRP, D-dimer and LDH concentrations, and WBC and neutrophil counts.

**Table 3.** The correlations between demographic, clinical, laboratory characteristics, and SARS-CoV-2 RT-qPCR Ct values in saliva samples and nasopharyngeal swabs.

Characteristics	Ct values from SS N = 61		Ct values from NPS N = 73	
	r/rho	*P*-value	r/rho	*P*-value
Age, yr	0.055	0.338	0.187	0.057
NEW score*	0.278	**0.015**	0.101	0.199
Charlson comorbidity index*	0.085	0.258	0.098	0.204
Interleukin-6, pg/ml	0.029	0.414	0.124	0.148
Ferritin, ng/ml	0.305	**0.008**	0.170	0.076
CRP, mg/dl	0.317	**0.006**	0.198	**0.047**
WBC count, per mm^3^	0.327	**0.005**	0.304	**0.005**
Neutrophil count, per mm^3^	0.330	**0.005**	0.321	**0.003**
NLR	0.151	0.123	0.116	0.164
Lymphocyte count, per mm^3^	0.256	**0.023**	0.168	0.077
D-dimer*, ng/ml	0.334	**0.004**	0.333	**0.002**
LDH, IU/l	0.195	0.066	0.284	**0.007**
Number of days since onset of symptoms	0.477	**<0.001**	0.411	**<0.001**

Abbreviations: CRP, C-reactive protein; Ct, Cycle-Threshold; LDH, Lactate dehydrogenase; NEW, National Early Warning; NLR, neutrophil to lymphocyte ratio; r, Pearson correlation coefficient; rho, Spearman rank correlation coefficient; * indicates variables compared by using Spearman rank coefficient; Significant values are shown in bold.

### Salivary SARS-CoV-2 Ct value as an independent predictor of severe COVID-19

Univariate and multivariate Cox proportional hazards regression analyses for prognosis of severe COVID-19 infection are given in Table 4. The results of univariate analyses showed that age, NEW score, Charlson comorbidity index, IL-6, ferritin, and CRP concentration, WBC and neutrophil count, NLR, LDH on admission, and Ct values ≤30 from SSs were associated with severe COVID-19 (Table 4). Ct values of NPS were not a prognostic factor for disease severity. In multivariate analysis, only Ct values from SSs retained significant prognostic value and therefore were the independent predictors for severe COVID-19 (Table 4).

**Table 4.** Univariate and multivariate Cox proportional hazards regression analyses for prognosis of severe COVID-19 infection.

Characteristics	Univariate analysis		Multivariable analysis	
	HR (95% CI)	P-value	HR (95% CI)	P-value
**Age** ≥ 65 years	2.832 (1.196–6.703)	**0.018**	0.617 (0.036–10.468)	0.738
**Gender** Male	0.614 (0.343–1.098)	0.100		
**NEW score** ≥5	5.035 (1.218–20.821)	**0.026**	1.035 (0.918–10.621)	0.977
**CCI** ≥3	3.589 (1.516–9.822)	**0.005**	4.029 (0.288–56.408)	0.301
**Interleukin-6** >55, pg/ml	2.983 (1.168–7.619)	**0.022**	0.380 (0.080–1.799)	0.222
**Ferritin** >500, ng/ml	2.170 (1.124–4.189)	**0.021**	1.908 (0.424–8.591)	0.400
**CRP** >100, mg/dl	2.596 (1.208–5.577)	**0.014**	0.551 (0.114–2.669)	0.459
**WBC count** >6.0, per mm^3^	2.669 (1.451–4.912)	**0.002**	0.562 (0.037–8.490)	0.677
**Neutrophil count** >4.0, per mm^3^	4.349 (2.272–8.325)	**<0.001**	3.862 (0.279–53.406)	0.313
**NLR** >5.94	5.660 (2.215–14.464)	**<0.001**	5.576 (0.457–70.569)	0.177
**Lymphocyte count** ≤1.1, per mm^3^	1.310 (0.733–2.341)	0.362		
**D-dimer** >500, ng/ml	1.872 (0.952–3.680)	0.069		
**LDH** >245, IU/l	2.346 (1.316–4.182)	**0.004**	1.463 (0,443–4.827)	0.532
**Ct values from SSs** ≤30	6.133 (1.841–20.428)	**0.003**	10.060 (1.835–55.140)	**0.008**
**Ct values from NPSs** ≤30	1.793 (0.956–3.363)	0.069		

Data are presented as hazard ratio (HR) and 95% confidence interval (CI). Significant values are shown in bold. All statistically significant variables in univariate analysis were included in multivariable analysis.

Abbreviations: CCI, Charlson comorbidity index; CRP, C-reactive protein; LDH, lactate dehydrogenase; NEW, National Early Warning; NLR, neutrophil to lymphocyte ratio; NPSs, Nasopharyngeal Swabs; SSs, Saliva specimens; WBC, white blood cell.

**Figure 5.**
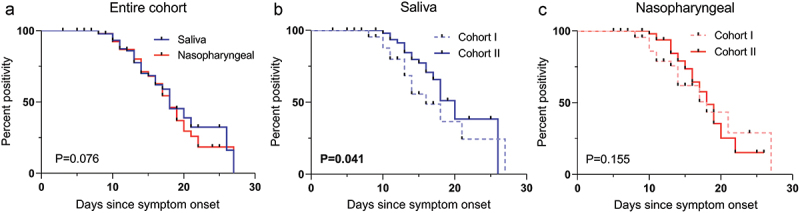
Kaplan–Meier analysis of positivity for SARS-CoV-2 prediction from saliva samples and nasopharyngeal swabs when the entire cohort (a) and mild to moderate illness (Cohort I) vs. severe disease (Cohort II) cases are analysed in saliva samples (b) and nasopharyngeal swab samples (c) separately.

In the Kaplan–Meier analysis, no significant difference was found in positivity for SARS-CoV-2 duration between SSs and NPSs when the entire cohort of 100 was analysed (median: 15.0 days *vs*. 17.0 days, *P* = 0.076) (Figure 5a). The median duration of SSs positivity in Cohort II was significantly longer than in Cohort I by the log-rank test (16.0 days [CI, 15.2–16.7 days] *vs*. 14.0 days [CI, 12.0–15.9 days]; *P* = 0.041) (Figure 5b). However, there was no difference in positivity duration in different COVID-19 disease severity groups using NPSs (16.0 days [CI, 14.5–17.5] *vs*. 17.0 days [CI, 14.1–19.8]; *P* = 0.155) (Figure 5c).

## Discussion

Quantitative reverse transcription polymerase chain reaction (RT-qPCR) is considered a gold standard confirmatory test for coronavirus disease 2019 (COVID-19). However, the relationship between SARS-CoV-2 Ct values which are directly related to the viral load and the severity of the disease is not fully understood and there is still a lack of tools for timely disease severity prediction. The most commonly used NPSs are uncomfortable and require trained healthcare staff to perform. There is also a risk of generating aerosol, which increases the viral transmission risk in healthcare workers [4]. Saliva samples (SSs) may be obtained safely and are compatible with self-collection. A number of studies with SSs revealed their suitability for the SARS-CoV-2 RNA detection [6–12,17–21]. The performed meta-analyses suggest that SARS-CoV-2 detection using SSs is of similar sensitivity or slightly less sensitive than NPSs [10,22]. Our study supported that SSs are a suitable source for COVID-19 diagnosis and even have some advantages over NPSs.

One of our main findings is that SS Ct values, unlike NPS, are associated with the severity of COVID-19 disease. Although conflicting, there are data that shows that NPSs Ct values are not associated with the severity of the disease [23–26]. We found no differences in NPSs Ct values in mild or severe disease in our study also. However, SSs SARS-CoV-2 RT-qPCR test became negative earlier in mild/moderate disease compared to severe disease (11.7 *vs*. 14.8 days), while there was no such difference in NPS tests. Even more, we found that Ct ≤ 30 from SSs were the independent predictors for severe COVID-19, while the virus load in NPSs was not a prognostic factor for disease severity. According to our data, we may suppose that in the case of mild/moderate disease, SARS-CoV-2 RT-qPCR test in SSs becomes negative earlier than in NPSs; however, in the case of severe COVID-19 infection, the SS RT-qPCR Ct values stay lower for a longer time. There are not many studies analysing SS SARS-CoV-2 association with the disease severity. Aydin et al. found that the saliva viral load in the early stages of COVID-19 infection may have a prognostic value in predicting disease progression in patients over 45 years of age [20]. Similar results were presented in a study where the mild disease pattern showed the high initial viral load in sputum and saliva after deep cough specimens in the first week with a peak in the viral load during the second week, followed by a decrease, and the severe disease pattern showed high levels even in the third and fourth weeks [27]. More studies are needed, but it seems that longer-lasting low Ct values in saliva could help to suspect a severe form of COVID-19 disease. Therefore, patients with low Ct values of simple saliva SARS-CoV-2 testing could be monitored more carefully, enabling timely hospitalization and administration of appropriate treatment measures by healthcare professionals.

Other interesting finding was that viral load reflected by Ct values in SSs was significantly higher than in NPSs, regardless of the severity of the disease. Low Ct value designates an elevated concentration of genetic material reflecting a higher viral load, typically correlated with a high infection risk [28]. The high value of Ct specifies a low concentration of viral genetic material with less infectivity risk; however, higher values may be seen in a later convalescent stage [29]. Both SSs and NPSs RT-qPCR Ct correlated clearly with duration of the disease in our study, with lower values in the first tests and higher in the third tests. Studies show [20,30,31] that the ACE2 receptor-rich minor salivary gland ductal epithelium and oral mucosal epithelium are the early targets for SARS-CoV-2 infection, and this fact could explain the high viral load in saliva at the early phases of infection. Thus, we may conclude that saliva samples are reliable materials for screening and detection of SARS-CoV-2.

The relationship between SARS-CoV-2 virus load determined by RT-qPCR and biochemical or haematological markers of COVID-19 disease is being extensively investigated; however, the data are ambiguous. Some studies [19,32–35] suggest associations between lower Ct values on admission samples and disease severity markers, including elevated levels of IL-6, LDH, CRP, lymphopenia and an elevated neutrophil/lymphocyte ratio. Interestingly, our data showed the opposite phenomenon – the positive correlation of higher LDH, D-dimer, CRP, leukocyte and neutrophil levels with higher Ct values in NPSs and ferritin, CRP, leukocytes and neutrophil levels in SSs, and obvious correlation with the number of days since the onset of the disease for both SSs and NPSs. However, there was a positive correlation in SSs (but not NPSs) between lymphocyte count and Ct values, with lymphopenia observed more often in the cases of higher viral load in saliva. Chua et al. in the paediatric COVID-19 study also found that children with lymphopenia were more likely to have a higher SSs viral load, but not NPSs viral load, which could support the interaction between COVID-19 infection, especially via the salivary glands, and the evasion of the immune system [36]. The discrepancies in associations between Ct values and biochemical or haematological markers between studies should be interpreted with caution because there is a wide heterogeneity in fluid samples collected at different phases of the disease. Our data showed that despite the differences in inflammatory and haematological markers, the mean Ct values in either SSs or NPSs were similar in severe disease or mild/moderate patient groups. However, there were more patients in the severe disease group included in our study at a later time and more patients in the mild/moderate disease group included in our study earlier. We may suppose that heightened inflammatory markers in severe patient group were influenced by the later stage of the disease, when hyperactive inflammatory response was involved in the disease process more than the virus by itself.

Despite the relevance of the data, there are some limitations of our study. The protocol of the study did not provide the inclusion of all subjects on a specific day of illness, so that the dynamics of Ct, the association with disease severity or laboratory parameters could be more accurately assessed. Consequently, despite the different clinical course, the differences in Ct values between the two cohorts could have been influenced by the time difference in symptom onset and sample collection. The inequalities between the positive corresponding NPT and SS samples (positive NPT, but negative SS and vice versa) might have also affected the final result. Another drawback to look out for is that RT-qPCR results cannot distinguish between viable and non-viable virus and reflect the total amount of viral RNA. Moreover, the SARS-CoV-2 quantification was assessed only in Ct values, not in viral load, which could have also limited the evaluation of active disease.

## Conclusions

Our study supports evidence that salivary SARS-CoV-2 RNA RT-qPCR testing is a valuable diagnostic tool for COVID-19 showing that cycle threshold values ≤30 are associated with severe COVID-19. Moreover, long-lasting positivity in salivary SARS-CoV-2 RNA RT-qPCR testing provides a longer window for the severe COVID-19 diagnosis, enabling healthcare professionals to select appropriate treatment options for patients.
